# Progress in Phototherapy

**Published:** 1902-11-29

**Authors:** 


### Progress in Phototherapy.

(*Concluded from page* 130.)

To control this reaction Schiff and Rosenthal[5] recommend the insertion of spent X-ray tubes in the circuit, or an automatic sparking arrangement. Holzknecht[6] has made a radio-chronometer for estimating the dosage of the rays, based upon the property that certain salts possess of turning blue when exposed to the rays.

Scholtz[7] gives the following list of diseases in which X-rays are said to be beneficial :—Lupus, lupus erythematosus, sycosis barbæ, favus, trichophytosis, hypertrichosis, acne, psoriasis, eczema, prurigo, alopecia areata, various forms of nævi. But with regard to this widely-differing list of complaints the indications for the employment of the rays and the manner in which the peculiar reactionary changes are brought about are still matters of dispute. Soft tubes, he states, give off more Roentgen rays than hard, and these rays are the only ones of therapeutic value given off by the tube. The maximum reaction is not reached for several weeks. This reaction is not only found at the site of entrance but also at the site of exit, the skin being affected more than the underlying tissues. Scholtz considers that the bactericidal action of X-rays is insignificant and therapeutically absolutely negligible. As evidence for this he states that he has exposed agar plates inoculated with virulent bouillon cultures of typhus, cholera and pyocyaneus, which had been covered with lead foil which had been pierced at one spot. The rays were directed upon this spot for upwards of an hour and the micro-organisms continued to grow in spite of the exposure. He has examined the skins of various animals after exposure to the rays. Pigskin after six or seven days showed distinct changes. The hair fell out and microscopically showed swelling and œdema of the epithelial cells with clumping and shrinking of the nuclei, while vacuoles appeared here and there in the protoplasm of the cells. Many nuclei appeared to be dividing without mitotic figures. The corium was markedly œdematous, its fibres swollen and badly stained. Elastin proved more resistent than collagen. The connective tissue cells were similarly affected to those of the epidermis, while the specialised epithelium of the sweat coils, hair follicles, etc., with the cells of the intima of the larger blood-vessels, showed the same condition. Around the blood-vessels were seen many inflammatory infiltrating cells, chiefly leucocytes ; masses of these showed beneath the epidermis and extended between the degenerated cells. The mast cells in the corium were increased. The centre of the lesion showed superficial vesicles, and these on breaking produce the ulceration. He concludes from these observations that (1) There is slow degeneration of cellular elements of the skin both epidermal and mesoblastic of the nucleus as well as protoplasm ; in this degeneration the fibrous elements elastin, collagen, as well as the muscles share. (2) Cell degeneration having reached a certain stage inflammatory reaction ensues ; the blood vessels dilate, serum and leucocytes are extravasted, the latter acting as phagocytes to destroy the degenerated cells. In lupus tissue he found the cells swollen, a large number of leucocytes, especially the polynucleated ones, between the plasma cells. A number of exceptionally large giant cells, with 100 to 200 nuclei, appeared to be the result of the ray exposure. These degenerated like the rest on inflammatory reaction setting in.

Carl Beck[8] divides the changes in the skin after *prolonged* exposure to the X-rays into three stages. (1) Hyperæmia—the cutis infiltrated and temperature raised, exfoliation, dilation of capillaries, and retrogressive changes in the differentiated elements of the skin, viz., glands, hair, nails. (2) Blisters—the corneous layer raised from the mucous stratum of the rete. The inflammatory signs well pronounced. (3) Escharotic destruction. The scabs on separating leave an ulcer behind, which may take months to heal. The latent period of this may be from 10–14 days, the more severe forms taking longer than the slighter. As a result of exposure to the rays the writer found on his hands, first, erythema, then terra-cotta pigmentation, then exfoliation in scales, and then the skin took on an appearance of leather, the perspiration ceased entirely, giving acute distress in hot weather. The roots of the hairs are destroyed. The curative influence on carcinomatous material cannot be doubted. Colloidal changes have been noticed in a case of adenocarcinoma treated by the rays, the adenoid characters having disappeared. A case of melanosarcoma is claimed by the writer to have been cured.

Rinehart[9] quotes Holland[10] as having cured a case of chronic eczema of the back of the hand with five applications of the rays, and Mackary as having cured two cases of eczema, while Gassman and Schenkel[11] he quotes as reporting a case of non-parasitic sycosis cured.

Stopford Taylor[12] reports the unique case of a man, aged 59, the subject of lupus erythematosus of the sebaceous variety on both cheeks and nose, who had in 1892 a typical epithelioma of the lower lip. This was excised. In 1893 there appeared an epitheliomatous ulcer on a horny excrescence on the tip of the nose. This was removed by potassa fusa (*Brit. Journal of Dermatology*, Jan. 1900). On January 10, 1901, there was a soft cauliflower growth on the whole surface of the nose. This was scraped, and the raw surface was subjected to the X-rays, 10 minutes daily at first and then 10 minutes twice weekly, with ung. Ac Salicyl gr. x. ad. ℥i. and lotio rub. as applications. The whole healed entirely, and is regarded as a cure.

[4] Amer. X-ray Jour., Nov. 1898. [5] Brit. Med. Jour., Oct 11. [6] Ibid. [7] Brit. Jour. Dermatology, Oct. 1902. [8] Ibid. [9] Amer. Jour. Med. Sci., July 1902. [10] Brit. Med. Jour., pp. 15. 99. [11] Fortschritte a.d. Gebiete Roentgen Strahlen, p. 1. [12] Brit. Med. Jour., May 3.

